# Chronic and Recurrent Diseases of the Skin in Relation to the Heart and Circulation

**Published:** 1914-01

**Authors:** 


Chronic and Recurrent Diseases of the Skin in Relation to the Heart and Circulation. David Walsh, M. D., Edin., senior Physician, Western Skin Hospital; London, England, *Medical Review,* September, 1913, concludes a clinical article as follows:

1 That a large number of chronic and recurrent skin eruptions are associated with disease of the heart.

2. That latent or unsuspected heart trouble is comparatively common amongst patients attending a skin hospital.

3. That prognosis in skin diseases in many instances depends on the state of the heart, and that attention to the state of the heart is imperatively demanded in a large number of skin affections.

4. That the circulatory inadequacy which apparently determines the onset and duration of many chronic and recurrent skin eruptions may likewise affect internal organs in a similar manner.

5. That what is called "idiosyncrasy" may in many cases simply describe a pathological instance of healthy reaction to moderate traumatism due to circulatory defect. In this way the irregular incidence of trade and drug eruptions may in many cases be accounted for predisposition being in such cases merely another name for central circulatory inadequacy with its connected disturbance of the balance of capillary circulation.

6. That in all cases of delayed healing of the skin from moderate traumatism of any kind, e. g., mechanical, thermal or septic, the state of the heart and circulation should be carefully investigated.

7. That in some cases the development of an acute cutaneous malady, such as a sun dermatitis, may afford delicate clinical evidence of failure of cardiac compensation.

8. That a similar process of impaired or pathological reaction to traumatism may be duplicated in the deep tissues of the body as well as on the visible skin, and that inadequacy of the capillary circulation may explain the development of various acute, chronic and recurrent diseases of internal organs following internal traumatism (cancer may possibly fall within this category).

9. That the delayed healing of wounds and the persistence of secondary traumatic complications may depend ultimately upon central circulatory defects, and may thus yield clinical evidence of heart disease with failing compensation not recognized by the text-books.

10. That where it is proposed to administer any drug, such as mercury or arsenic, which is known to act as a tissue irritant, the possession of a normal or a fully compensated central circulation should be made a *sine quâ non.*

11. That the dermatologist who wishes to do justice to himself and to his patients should keep an eye upon the heart, as there is evidence to show that many diseases of the skin may be materially modified as to diagnosis, prognosis and treatment by their relation to the circulation.

